# Hierarchy of fillings for the FQHE in monolayer graphene

**DOI:** 10.1038/srep14287

**Published:** 2015-09-22

**Authors:** Patrycja Łydżba, Lucjan Jacak, Janusz Jacak

**Affiliations:** 1 Faculty of Fundamental Problems of Technology, Wroclaw University of Technology, Wyb. Wyspiańskiego 27, 50-370, Wrocław, Poland

## Abstract

In this paper, the commensurability conditions, which originated from the unique topology of two-dimensional systems, are applied to determine the quantum Hall effect hierarchy in the case of a monolayer graphene. The fundamental difference in a definition of a typical semiconductor and a monolayer graphene filling factor is pointed out. The calculations are undertaken for all spin-valley branches of two lowest Landau levels, since only they are currently experimentally accessible. The obtained filling factors are compared with the experimental data and a very good agreement is achieved. The work also introduces a concept of the single-loop fractional quantum Hall effect.

The fractional quantum Hall effect (FQHE) in classical semiconductor structures has been widely examined theoretically^1^,^2^,^3^,^4^,^5^,^6^,^7^ and many experimental results – even with a depiction of fractional filling factors out of the lowest Landau level (LLL) – have been provided^8^,^9^,^10^,^11^. Recenty, a lot of interest has been focused on the FQHE appearing in the monolayer graphene samples^12^,^13^,^14^,^15^,^16^,^17^,^18^,^19^,^20^,^21^,^22^,^23^,^24^. This enthusiasm arose from a very specific character of the graphene energy spectrum. Its gapless nature and a nonzero Berry phase^23^,^25^ cause the lowest Landau level to be placed exactly in the Dirac point, which connects the valence (VB) and the conduction band (CB). Thus, the LLL is equally shared between free electrons and free holes. This year, a paper reporting fractional ratios reaching fifth spin-valley branch (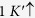 subband of the first Landau level) has been published^24^. The very popular Jain’s model of composite fermions (CFs – complexes of electrons and magnetic field flux quanta pinned to them)^4^,^5^, despite of its obvious artificial character, can provide a pretty good qualitative analysis of the FQHE in the lowest Landau level of graphene. For example, the hierarchy of fillings can be established and the Laughlin correlations can be introduced with the use of the Aharonov-Bohm effect^26^. However, this model seems to be insufficient when it comes to the higher Landau levels (LLs). Noticing that not all filling factors can be derived in the the monolayer graphene case creates an impression that this simplistic single-particle model cannot embrace the entire physics of the quantum Hall effect systems. For this reason, in this article, we introduce the cyclotron subgroups model^1^,^2^,^3^, which is based on the topology of two-dimensional systems upon a strong magnetic field presence, to the issue of the FQHE in graphene.

The main part of this paper (the third section) is divided into separate subsections, each concentrating on a different sublevel of the given Landau level (enumerated with a classical electron spin and a valley pseudospin called isospin). For every spin-valley branch appropriate commensurability conditions are applied. These terms allow to determine filling factors for which the cyclotron orbit of an arbitrary electron fits perfectly to the interparticle distance and exchanges of particles as well as the collective quantum Hall states can be realized. Solely the zeroth and the first Landau level (1LL) are experimentally accessible and for this reason only these are analyzed in this paper.

## Results

### The application of commensurability conditions

Before proceeding to the application of commensurability conditions, one should realize that there is a great difference between a Hall state in graphene compared to one for a two-dimensional electron gas (2DEG) in a conventional semiconductor – even when the filling factors are equal^22^. In the latter case, the filling ratio *ν*_*semi*_ is measured in relation to the empty LLL. However, in graphene, since a half of the zeroth Landau level is placed in the VB (due to the Berry phase), *ν* is counted with respect to the first empty sublevel placed in the CB (a third spin-valley branch of the LLL). This change in the terminology arises from the fact, that it is natural to define *ν* in terms of an electronic density measured from the Dirac (neutrality) point, so from the bottom of the conduction band^25^. Thus, a zero filling ratio in graphene *ν* = 0 actually corresponds to two completely filled spin-valley sublevels – 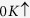 and 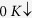. Finally, the relation between graphene and typical semiconductor filling factor can be expressed in a form,


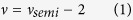


**Figure 1:**
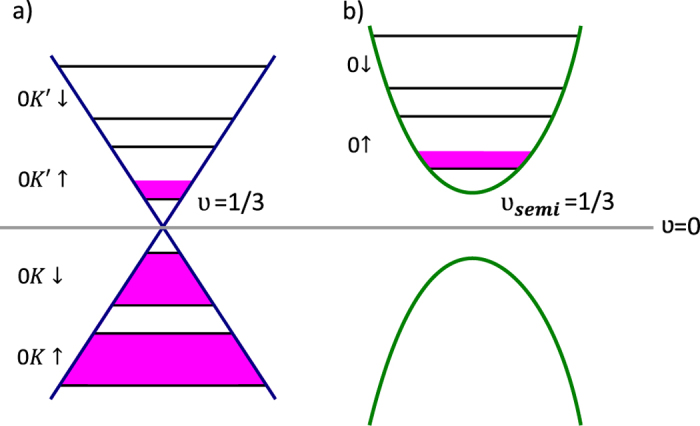
The comparison of a 1/3 fractional Hall state for a monolayer graphene (**a**) and a typical 2DEG (**b**). The pink colour symbolizes filled (with electrons) parts of subsequent sublevels of the LLL. In a classical two-dimensional semiconductor the filling factor *ν*_*semi*_ is measured in relation to the empty lowest Landau level. However, in graphene, since a half of the lowest Landau level is placed in the VB (due to the Berry phase), the filling factor *ν* is not counted with respect to the empty LLL, but with respect to the first empty sublevel of the LLL placed in the CB (third spin-valley branch of the LLL called 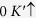). Thus, such filling in graphene 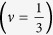, unlike in a traditional semiconductor, is accompanied by two completely filled sublevels of the LLL located in the VB^22^,^25^. The relation between graphene and typical semiconductor filling factor can be expressed in a form *ν* = *ν*_*semi*_ − 2.

For example, 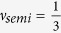 filling for a traditional 2DEG corresponds to 
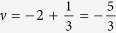
 for graphene samples (Fig. 1).

Let us remind, that the effective low energy Hamiltonian for a monolayer graphene is equal to^23^,


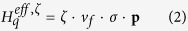


where *ζ* = ±1 (plus for *K* Dirac point and minus for *K*′ Dirac point), *σ* is a vector of Pauli matrices (connected with sublattice pseudospin) and **p** is a quasimomentum. Note, that if one express the quasimomentum with its length and direction **p** = *p***n**, then the Hamiltonian can be rewritten in the form,


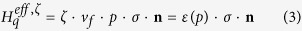


where, **n** = (*ζcos*(*Jϕ*), *ζsin*(*Jϕ*)) (for a monolayer graphene *J* = 1, but it is convenient to introduce this parameter at this point, because after applying *J* = 2, the obtained 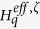 is proper for a bilayer graphene samples^27^), *ε*(*p*) is the absolute value of the energy eigenvalue. The operator *σ* ⋅ **n** projects the pseudospin onto the direction of the momentum **p**. Note that the eigenstates of the Hamiltonian are also eigenstates of this projection operator with eigenvalues +1 for electrons and −1 for holes. In conclusion, the Dirac particles are chiral and the pseudospin is always parallel to the momentum for free electrons from conduction band and always antiparallel for free holes from the valence band^23^,^28^. Simultaneously, the form of the eigenfunction (spinor),


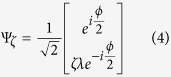


where *λ* = ±1 (plus for the CB, minus for the VB) and *ϕ* = *tg*(*p*_*x*_/*p*_*y*_) minus 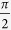 stands for the angle between the quasimomentum and the abscissa. During the adiabatic evolution of such state, the quasimomentum (and so the vector *n*) is rotating through *ϕ* in the reciprocal space. When the quasiparticle encircles a closed contour in the momentum space (*ϕ* = 2*π*), the eigenfunction acquire a nonzero phase equal to *π*, called the Berry phase,


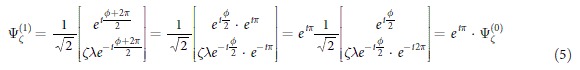


This feature should not be surprising, since graphene particles are chiral and a rotation of the momentum leads to a rotation of the sublattice pseudospin (in the case of the typical free electron spin a full rotation also leads to a *π* phase gain of the eigenfunction). Furthermore, the Berry phase (and the *J* parameter) defines the degree of degeneracy of the LLs^27^. For a monolayer graphene 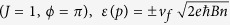 and only *n* = 0 Landau level lies at zero energy, while for a bilayer graphene 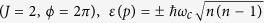 both *n* = 0 and *n* = 1 lie exactly at the neutrality point. Thus, the Berry phase gives rise to a unique form of the lowest Landau level in a monolayer graphene, which is placed exactly at the Dirac point. For this reason, the LLL has states (organized in four sublevels) distributed equally between the CB and the VB. Finally, filling factors for free holes from the valence band are just a mirror reflection – with a minus sign – of filling factors for free electrons from the conduction band, therefore it is convenient to determine *ν* in relation to the bottom of the CB.

**Figure 2:**
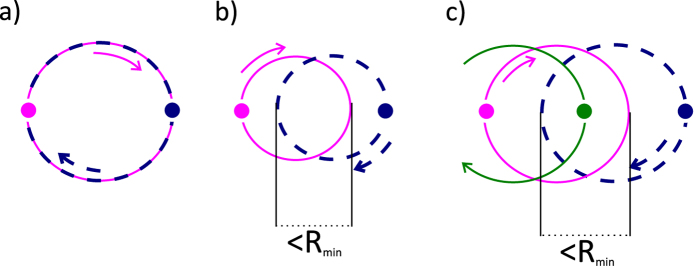
This figure presents electrons placed on a 2D manifold in the presence of a strong magnetic field. The exchanges of neighbouring particles are possible only when the cyclotron orbit fits perfectly to the interparticle distance (**a**). When the cyclotron orbit is too short the exchanges are not allowed, since the condition of maintaining the minimum distance *R*_*min*_ between fermions (protected by Coulomb repulsion forces) is not obeyed (**b**). Similar situation appears when the cyclotron orbit is larger than the spacing between electrons. However, in this case, the condition is broken not for nearest but (for example) for next-nearest neighbours (**c**).

In order to apply the topology based commensurability conditions^2^,^3^ to graphene, let us remind that this conditions originated from the necessity of determination of generators of the braid group describing exchanges of particles on a plane. Upon the magnetic field not all trajectories are available, what leads to a redefinition of the full braid group^1^. Only if cyclotron orbits fit to the interparicle separation, the mutual exchange of neighboring particles on a 2D (two-dimensional) manifold with uniform particle distribution is possible (Fig. 2). Otherwise the cyclotron orbits are too short to match closest particles or too large to maintain a particle separation fixed by the uniform density (rigidly kept constant by Coulomb interactions). The cyclotron orbits have the same size for all particles due to a flat band condition reducing the kinetic energy competition and resulting in the same averaged velocity in a presence of a perpendicular magnetic field (though quantumly, velocity is not well defined since its coordinates do not commute). The cyclotron orbits restrict the topology of all trajectories uniformly in 2D, thus restrict the braid group structure despite of particularities of an interaction and other fields, e.g. of the crystal field. Therefore, for graphene, the cyclotron orbit structure is governed by ordinary Landau Levels restrictions despite a specific band structure with Dirac points. The latter particularities of graphene quantum dynamics are included in the ordinary part of the Feynman path integral, whereas additional summation over topologically nonequivalent trajectory classes concerns the braid group structure the same as for a 2DEG upon the magnetic field. *The difference between the conventional 2DEG systems and graphene will be here related with distinct degeration of LLs in graphene compared to a typical semiconductor 2DEG.*

The kinetic energy of electrons located on a graphene sample upon a strong magnetic field perpendicular to its surface can be thus established from the Landau level quantization (the crystal field does not affect the characterisation of the kinetic energy),


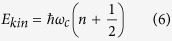


where 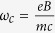 is the cyclotron frequency and *n* = 0, 1, 2… enumerates the Landau levels. The cyclotron orbit size is proportional to *E*_*kin*_ and clearly depends on *n* numerating LLs, thus it has a distinct value for electrons from different LLs. The coincidence of a cyclotron orbit and an orbit that embrace a single quantum of an external magnetic field flux in the case of completely filled LLL, allows to use this orbit area as the definition of the cyclotron orbit size.

In a graphene all LLs splits into four sublevels with the same degeneration and distinction of electrons due to the ordinary spin of electrons (↓, ↑) and the valley pseudospin (*K*, *K*′). This sublevel degeneration equals to,


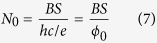


where, *S* represent the sample surface and 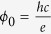 is a quantum of the magnetic field flux. Preceding subsections contain a derivation of the fillings *ν* which give rise to integer and fractional quantum Hall states, based on an assumption that such collective state can be realized only when particle exchanges are possible (as required for determination of the statistics for a correlated multiparticle state), i.e., when the cyclotron radius matches perfectly to the integer multiple of a half of minimal distance between particles (protected by Coulomb repulsion forces).

### Completely filled subband 0 *K*′↑ of the lowest Landau level

In the case of a completely filled 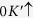 sublevel of the LLL, the number of particles is equal to the value of the degeneration,


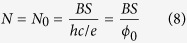


From the above equation it is easy to conclude that also the number of quanta of an external magnetic field flux equals *N* = *N*_0_ and so the minimum area per particle embraces one *ϕ*_0_. For this reason, we can establish the surface of the cyclotron trajectory,


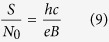


We assumed that for *ν*_*e*_ = 1 the loopless exchanges (described with full braid group generators) are possible.

### Partially filled 0 *K*′↑ sublevel of the lowest Landau level

After the applied magnetic field is raised (*B* > *B*_0_, where *B*_0_ corresponds to the completely filled lowest sublevel of the LLL), the number of electrons filling the 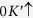 Landau sublevel becomes smaller compared to the degeneracy *N* < *N*_0_. Therefore, the cyclotron radius is not long enough to reach the neighboring carrier and the statistic cannot be determined. To create a collective multiparticle state (like a fractional quantum Hall state) the cyclotron radius must be enhanced by using braids of multi-looped type (as has been proved^1^, in 2D spaces multi-looped braids can match neighboring particles and substitute single-looped generators of the braid group),


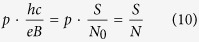


where *p* is an odd number (a half of a closed, *p*-looped cyclotron trajectory needs to form a proper, *open* exchange trajectory, thus *p* cannot be even). Simple transformations allow for determining the *N* to *N*_0_ ratio – the filling factor for particles,


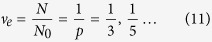


and analogically for holes in this sublevel (*let us emphasize that they are not free holes from the VB in graphene, which due to a particle* – *hole interband symmetry in Dirac points may be assigned with the same fractions as free electrons from the conduction band with sign reflection only*),


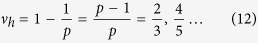


The generalized hierarchy, similarly as in a 2DEG^1^, can be established if one assumes that only *p* − 1 loops of a cyclotron trajectory embrace exactly one external magnetic field flux quantum each, whereas the last one embrace only a fraction of *ϕ*_0_, but equal to the number of quanta per particle calculated formally for other (even fractional) fillings,


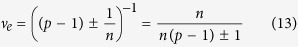



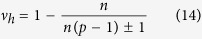


Thus *n* does not need to be an integer number (like in the Jain’s hierarchy^4^,^5^) – it might be equal to other fraction *ν* (this leads to a fractal-like construction). Examples of the above filling hierarchy for electrons are presented in the Table 1.

Fillings with even denominators (like 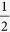) can also be established from the presented picture, if one assumes that the last loop takes zero flux quanta *ϕ*_0_ and describes a free movement like in a Fermi liquid without an external magnetic field *B* (it is equivalent to the limit *n* → ∞),


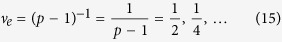



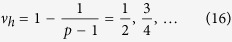


This compressible liquid states are referred to as the Hall metal states.

### Filling of the 0 *K*′↓ sublevel of lowest Landau level

For a decreasing magnetic field (*B* < *B*_0_) the second sublevel of the LLL is being filled with *N* − *N*_0_ free carriers (remaining *N*_0_ particles must be located in the lowest 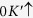 sublevel). The cyclotron orbit with the size as before 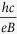 does not fit to the particle distribution,


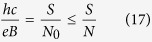


Only in one exceptional situation the cyclotron radius is long enough to reach neighbouring particles,


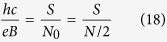


The resulting integer filling factor *ν* = 2 corresponds to both – 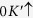 and 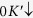 – completely filled (so experiencing integer quantum Hall effect – IQHE) subbands of the LLL.

If, however, the cyclotron orbits are shorter in comparison to the particle separation, the multi-loop trajectories are needed to restore the exchanges. In the *p*-looped case (*p* – odd),


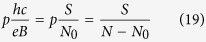


thus 
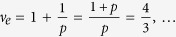
 and dually for holes 
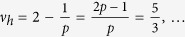
 Let us note, that the fractional quantum Hall states for the second sublevel are generated by less than half of electrons (the rest *N*_0_ participate in a creation of the IQHE in the lower subband), hence the dips in a longitudinal and plateaus in a transverse resistivity may not be so pronounced.

Analogically, assuming that only a fraction of a flux quantum falls on a last loop from the *p*-looped trajectory, we can generalize the hierarchy (Table 2),


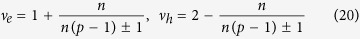


### Filling of the 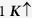 subband of the first Landau level

While the 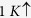 subband of the first Landau level is filling, the total number of free particles is raising from a double degeneracy 2*N*_0_ to a triple degeneracy 3*N*_0_ (for *ν*_*e*_ = 3). During this process, *N* − 2*N*_0_ electrons are being located on the 1LL and remaining 2*N*_0_ are experiencing the IQHE in the zeroth Landau level. *It may be misleading that we only include two sublevels of the LLL in our considerations, but remember that N denotes the number of free electrons in the conduction band and for that kind of particles only two sublevels are available. Additionally, the filling factors for free carriers from the VB (holes) are a simple mirror reflection (with a minus sign) of filling factors for free carriers from the CB (electrons).*

The surface of a cyclotron orbit from the first Landau level is considerably changed and equals to,


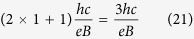


This situation (sudden growth of the cyclotron orbit size) allows for new possibilities – new allowed commensurability conditions, which are listed below^2^,^3^:

1. The cyclotron orbit may fit perfectly to the minimal interparticle separation in this subband,


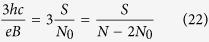


thus obtained filling factors are 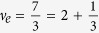 and analogically for holes 
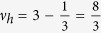
. *Note, that in this case multi-looped trajectories are not needed to form a Hall state in a fractionally filled Landau level, so obtained filling factors correspond to the single-loop FQHE. This new Hall feature is possible only for n* > 0. *Although the quantization of the transverse resistivity is fractional and simmilar to that for ordinary FQHE* 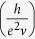, *the Laughlin correlations are represented with the p* = 1 *power in Jastrow polynomial, which displays single-loop braid exchanges similar to that in IQHE. The system is, hence, described with a full braid group. This specific FQHE state is associated with single-loop cyclotron braids, in contrast to the multi-loop braided FQHE, typical for n* = 0*. It seems, that the stability of this novel effect might be comparable to the stability of the IQHE, rather than of the ordinary FQHE, what was confirmed in* ref. 24 *and described in more details in a “Comparison with experiment” paragraph.*

2. Also in this band the cyclotron radius might be too short to match the distance between particles. Then the multi-loop trajectories have to be introduced resulting in the FQHE for electrons,


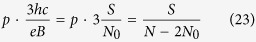


thus 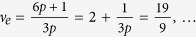 We can also estimate the dual filling factor for holes 
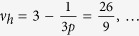


The generalized hierarchy can be established in the same manner as for subbands of the zeroth Landau level (we could also derive in that manner the Hall metal hierarchy, *n* → ∞),


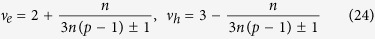


Exemplary fractions are presented in the Table 3. It is worth to emphasize, that in the Jain’s model the FQHE of electrons is described as the IQHE of CFs. Additionally, within this construction *n* stands for the number of completely filled Landau levels in a diminished effective magnetic field experienced by CFs, so it needs to be an integer number. However, in the discussed cyclotron model, 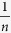 is a portion of a flux quantum that falls on the last loop of a multi-looped cyclotron trajectory (an implementation of a multi-looped braid). Therefore, 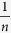 can be set equal to any number of magnetic field flux quanta per particle in the system that ensures, or rather allows, the creation of an collective Hall-like state. Finally, *n does not need to be an integer number*. We assume, that it can take the value of any fractional filling factor derived from the commensurability conditions. It is also worth to notice, that 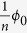 embraced by a cyclotron orbit from the LLL (defined with exactly one flux quantum), actually corresponds to 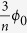 embraced by a cyclotron orbit from the 1LL (defined with exactly three flux quanta). Additionally, some other commensurability conditions are also worth to consider. The size of a multi-looped trajectory may match the separation of every *m*'th particle (the biggest value of *m*, equal to 3 in the 1LL, is established from a condition leading to the IQHE formation in this Landau band). Obtained hierarchies pretty outstandingly agree with experiment (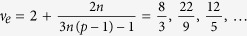

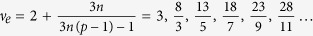
; 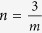; *m* – integer; *p* = 3).

We have also noticed that only fillings which are separated from the nearest integer *ν* by more than 0.3, are (at the moment) experimentally observable (in the table this filling factors are marked with a blue colour). Others, are lost in a deep and extended minimum of a longitudinal resistivity connected with the integer *ν*.

3. It is also possible that the cyclotron surface matches to twice the minimal interparticle separation, which allows every second carrier to exchange (it allows also for establishing of the statistics),


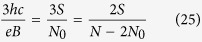


thus 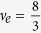 and 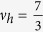 (also the single-loop FQHE states – no need for multi-looped trajectories).

4. The last condition is fulfilled when the cyclotron orbit fits the minimal separation of every third particle (making their exchanges possible),


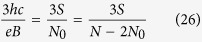


Finally, *ν* = 3, which corresponds to completely filled three subbands (each generating the IQHE) of Landau levels (two from the LLL – 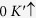 and 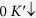; one from the 1LL – 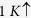).

### Filling of the 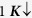 subband of the first Landau level

For low magnetic fields, when a number of particles is in the range 4*N*_0_ > *N* > 3*N*_0_, one deals with a filling of the 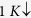 sublevel of the 1LL. In lower subbands particles are generating the IQHE, in subbands of the LLL (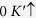 and 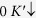) the cyclotron orbits fits perfectly to the electron separation 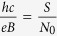, while in the 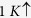 sublevel of the first Landau level the commensurability condition has the form 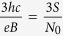.

In this subband there are *N* − 3*N*_0_ electrons, the cyclotron orbits have size 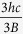 and the quantum Hall-like states can occur for filling factors derived from commensurability terms:

1. If the cyclotron orbit equals to the particle separation, the commensurability requirement,


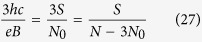


This condition is fulfilled, when 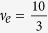 and dually for holes 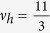 (the single-loop FQHE is obtained).

2. If the cyclotron orbit is too short to reach the neighbouring particles, then multi-looped trajectories need to be introduced. This happens for fillings,


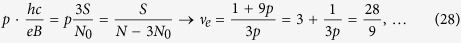


The conjugate filling factor (for holes) can also be estimated 
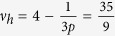
.

The generalized hierarchy takes the form of,


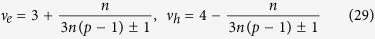


One should notice that the fractions obtained experimentally^24^ can be easily derived from the above equation, if one considers *n* equal to the filling factors presented in the Table 1. Exemplary calculations,


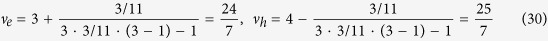



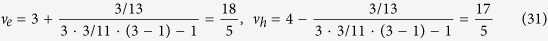


This fractions may also be accomplished with a use of commensurability conditions mentioned previously: 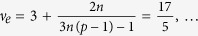; 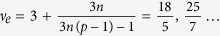; 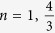; *m* – integer; *p* = 3.

3. For a commensurability condition 
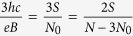
 – corresponding to the cyclotron orbit allowing for exchanges of every second electron – the filling factor is equal to 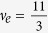 (and dually for holes 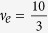).

4. Finally, a commensurability condition 
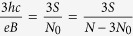
 (exchanges of every third electron) results in the IQHE for all four subbands (*ν* = 4).

### Filling of the 1 *K*′↑ subband of the first Landau level

Further reducing the strength of an external magnetic field results in four completely filled Landau sublevels (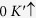, 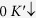, 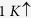, 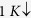) and a partially filled third (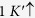) subband of the 1LL. In the subbands of the LLL the commensurability condition 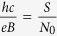 is fulfilled, while in 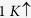 and 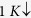 subbands from the 1LL the commensurability condition takes the form of 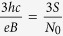. The total number of particles is placed between 4*N*_0_ and 5*N*_0_ and the surface encircled by a cyclotron orbit of a carrier from the 1LL is equal to 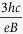. Here, there are following possibilities:

1. The cyclotron orbit may fit to the minimal distance between particles,


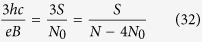


thus 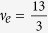 (and the conjugated state 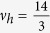) corresponds to the single-loop FQHE in a partially filled 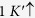 subband of the 1LL.

2. If the cyclotron radius is shorter than the particle separation, then multi-looped orbits are required resulting in the FQHE in this subband. Appropriate filling factors are determined from the condition,


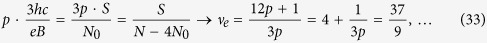


Symmetrically for holes 
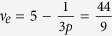
. Also this hierarchy can be generalized (assuming that on a last loop from a *p*-looped trajectory falls only a 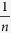 portion of a flux quantum),


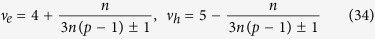


Exemplary (experimentally observable^24^) filling factors,


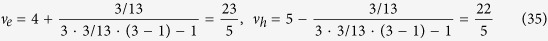


and


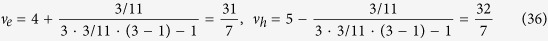


This fractions may also be accomplished with a use of commensurability conditions mentioned previously: 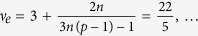; 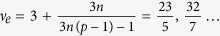; 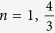; *m* – integer; *p* = 3.

3. It is possible that the cyclotron trajectory fits perfectly to the separation of every second particle and the commensurability condition 
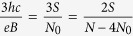
, and so the filling factor 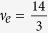 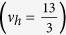 corresponds to the single-loop FQHE for a partially filled third sublevel of the 1LL.

4. Finally, a commensurability condition 
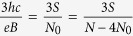
, *ν*_*e*_ = 5 corresponds to the IQHE in all five (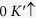, 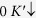, 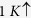, 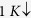 and 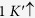) Landau subbands.

### Filling of th 1 *K*′↓ subband of the first Landau level

Partial and complete filling of the last subband of the 1LL can be achieved after reducing the strength of the magnetic field and attaining the region 6*N*_0_ > *N* > 5*N*_0_. As in all previous cases, lower sublevels are already completely filled, fulfilling all conditions for realization of the IQHE (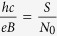 in the LLL and 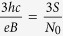 in the 1LL). The remaining *N* − 5*N*_0_ electrons are moving along the cyclotron trajectories (as braid group elements) that encircle a 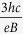 surface. It is worth to mention that fractions from the last subband of the 1LL are currently not experimentally accessible. All possible conditions and resulting *ν* are listed below:

1. The commensurability condition,


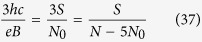


and filling factors 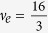, 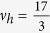 correspond to the single-loop FQHE in a partially filled last sublevel of the 1LL.

2. For a cyclotron radius shorter than the interparticle separation, the multi-looped trajectories need to be introduce and a commensurability condition takes a form,


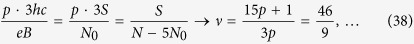


and dually for holes 
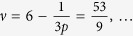
.

3. It is possible that the cyclotron trajectory fits perfectly to the separation of every second particle and the commensurability condition 
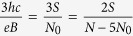
, 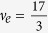 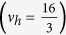 corresponds to the (single-loop) FQHE for a partially filled 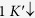 sublevel of the 1LL.

4. Finally, commensurability condition 
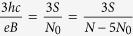
, *ν*_*e*_ = 6 corresponds to the IQHE in all five Landau subbands.

### Summarizing of filling ratios obtained with the use of the commensurability conditions

All of the filling factors obtained in preceding subsections – from various commensurability conditions – are captured in the Table 4. Note, that in a very similar manner as presented in the previous section, one can estimate the filling factors for higher Landau levels, only with the use of the commensurability conditions,

1. If the cyclotron orbit fits perfectly to the interparticle separation, the condition takes the form of,


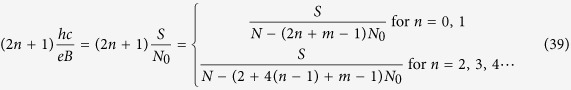


where, *n* = 0, 1, 2… is the Landau level index and *m* = {1, 2, 3, 4} (*m* = {1, 2} in the LLL) enumerates the sublevels of LLs.

2. If the cyclotron orbit is twice in dimension of the interparticle separation,


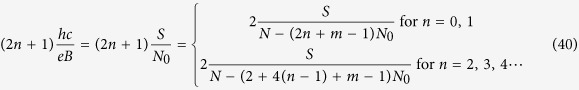


3. If the cyclotron orbit fits to the separation of every third particle,


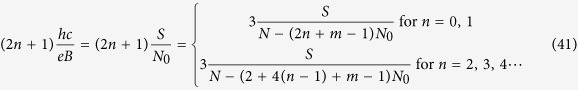


4. The last commensurability condition is useful in the case, when the cyclotron orbit is too short to reach the neighbouring particle and the multi-loop trajectories are necessary to restore exchanges,


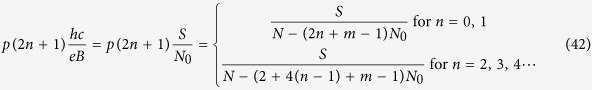


Note, that the generalized hierarchy can also be derived.

Also, one can define which *ν* are filling ratios of the ordinary FQHE and which of the single-loop FQHE. Note, that an integer quantum Hall effect can be defined as a collective state that is formed without the implementation of multi-looped trajectories (multi-looped braids) to provide the particle exchanges. In the same time, the ordinary fractional quantum Hall effect is defined as a collective state with multi-looped trajectories implemented. That is why it is rather improper and may be misleading to call the collective states described with fractional filling factors, but created with cyclotron orbits with no additional loops, as the ordinary FQHE states or even the IQHE states. In this paper we named this unique *ν* as single-looped FQHE filling factors.

It is also worth to emphasize, that the role of the Coulomb repulsion force is crucial even within the cyclotron subgroup model, since it ensures that the minimal interparticle separation (particles cannot approach closer to each other) is rigidly kept. Otherwise, it would be kept only in average and trajectories outside the cyclotron subgroup could be occasionally realized. Besides the interactions, also the two-dimensionality of a manifold is an important prerequisite (the rich structure of a full braid group). Finally, the commensurability conditions of the cyclotron orbit and the minimal interparticle distance reflect the necessity of exchanges of neighbouring particles for creation of collective states like the fractional (ordinary or single-looped) and integer quantum Hall states.

### Comparison with the experiment

Since its first isolation by Geim and Novoselov (2004), graphene was subjected to the intensive, experimental research. Due to very strong interactions between massless Dirac particles (as a result of small dielectric constant *ε* in comparison to a standard 2DEG) signs of a collective behaviour, resulting in the integer and the fractional quantum Hall effect formation, were highly expected. Also the possibility of modifying with a lateral gate voltage (*V*_*bg*_ <10 *V* to avoid collapse of a sample induced by electrostatic attraction^14^) of the carrier density in a fixed magnetic field strength, made experiments on monolayer graphene exceptional. Although the IQHE appeared to be extremly robust, allowing for its observation even in room temperatures^29^, the FQHE remained hidden by the cause of a high disorder level. A significant improvement in transport properties was achieved after a preparation of graphene both suspended and placed on a boron-nitride substrate. However, scientists experienced a great disappointment – measurements carried out on these samples in a standard Hall-bar geometry failed to develop quantum Hall features^30^ (a recently achieved^31^ implementation of non-invasive contacts, which are not strictly connected to the probed area of a sample, finally allowed for an observation of Hall-like states in four-terminal measurements). Later it was argued^16^, that the possible problem in multi-terminal devices may lie in small dimensions of samples (especially a small length to width ratio *W*/*L*). The voltage probing lead placed within a large potential drop region – a hot spot – and a proximity of the current probing lead may result in shorting out of the Hall voltage. Additionally, adsorbates may not be completely removed from a device body – but only redistributed over a monolayer graphene flake – upon the current annealing (passing a large current through a sample)^31^. This problem also arises from the interfering nature of electrodes in the Hall-bar geometry, which act like heat sinks – electrons with a very high kinetic energy can easily leak out through voltage probing terminals – and give rise to an inhomogeneous temperature profile^31^. Consequently, abandoning a typical measurment geometry and adopting a two-terminal observation metod allowed for the first observation of a 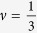 FQHE state in graphene^14^,^19^. One should, however, know, that a detailed description of a quantum Hall state in the two-terminal geometry can be quite tricky. For exemple, the value of obtained conductance *G* actually depends on both longitudinal and transverse conductivities^16^. Although, in a vicinity of *σ*_*xx*_ dips, the total conductivity *G* is expected to coincide with *σ*_*xy*_, its value may still be higher than those of traditional Hall-bar measurements^14^. Also the estimation of a FQHE excitation gap – and other quantitative characterizations – cannot be performed in a straightforward manner^18^. The latter requires an appropriate theoretic approach, like the Gaussian model with peak positions and widths, together with a sample *W*/*L* relation (effective rather than real, since its value may differ from the geometric aspect ratio of the device), as parameters^16^. It was also demonstrated, that in such devices pinning of an electron density below the contacts, which results in a inhomogenous density in the graphene sample and a p-n-p junction formation (or p-n, n-n’ and others), gives rise to a fractional conductance *G* connected with IQHE and not FQHE as expected^16^,^32^.

Moreover, recently other type of a measurement setup appeared to be very successful in developing of collective features – especially in the LLL. Since graphene flakes may be much cleaner on the nanometer scale, probing a smaller area can allow for observation of the fragile FQHE states, which are absent in typical transport experiments. Because of a nonzero disorder, the typical procedure gives only an average view of the sample characteristics and may cover up some interaction induced effects^17^. The microscopic information is not blurry and can be obtained with the use of local electronic compressibility measurements, performed with single-electron transistors^12^,^15^.

It is also worth to emphasize, that finally some plateaus in *σ*_*xy*_ corresponding to the integer and the fractional quantum Hall effect were observed in multi-terminal devices^18^,^22^,^24^. The progress in a Hall-bar measurements was achieved after replacing the invasive contacts, directly connected to a sample body probed by transport measurements, with the non-interfering electrodes, connected with a flake by the etched constrictions^31^. As a matter of fact, the experiment carried out in this geometry by Amet *et al.*^24^ was the first one to reveal the plethora of fractional quantum Hall states in the first Landau level (although a few states, mostly associated with the single-loop FQHE, were already evidenced earlier^22^).

Additionally, in high magnetic fields, in graphene samples, a transition to the insulating state was observed for low concentrations of carriers^14^,^19^. The latter manifest itself as a dramatic increase in the resistance. It was also pointed out by Du *et al.*^19^, that the fractional quantum Hall states (like *ν* = −1/3) may compete with this insulating phase, leading to the disappearance of Hall plateaus in the presence of high disorder. However, the FQHE may reappear after annealing of the sample. Thus, a great value of mobility seems to play a triggering role in a collective Hall-like state creation, what stays in perfect agreement with the cyclotron subgroup model, since a sufficiently longer mean free path is needed to traverse multi-looped trajectories.

The recent measurement of the transconductance in the PET configuration of small scrapings of graphene (0.8 × 3 *μm*) in a varying gate voltage *V*_*bg*_ + *δV*_*bg*_(*t*) (*f* = 433 *Hz*, *V*_*bg*_ ~ 1 *V*, *δV*_*bg*_ ~ 10 *mV*), has revealed a fine structure of local correlated states^17^. This structure is visible in typical transport experiments as noisy-like oscillations but in the not-noisy regime. The detailed inspection of this fluctuations was done by visualization of the transconductance in the (*N*, *B*)-plane, which revealed a highly ordered pattern (Fig. 2 in 17) attributed to series of local correlated states closely accompanying fractions for the IQHE (and the FQHE as well) in monolayer graphene with broken SU(4) symmetry. The linear character of this new feature was discovered, including several bunches collinear to directions of main IQHE/FQHE ratios in (*N*, *B*)-plane. Let us briefly investigate these peculiarities. Straight lines, which lie in a (*N*, *B*)-plane and have a common point in a coordinate origin, are connected to a constant filling factor value; 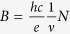. In ref. 17 units were selected in a manner, that 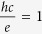. An arbitrary line in a (*N*, *B*)-plane can be described with an expression *B* = *αN* + *β*. Its successive points are related to filling factors making up the hierarchy 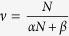, (*αN* + *β* = *N*/*ν*). The latter seems to match quite well the FQHE hierarchy in consecutive subbands of LLs, according to the scheme arising from the commensurability conditions for monolayer graphene in magnetic field presence, 
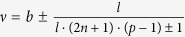
 with *n* enumarating Landau bands, *b* depending on *n* and level degeneracy, first ± arising from an electron – hole symmetry and second one – from the ‘eight-figure-shape’ of the trajectory. These fractions are gradually shifted towards subband edges with growing *n* and compressed to smaller periods for higher *n*, which also agrees with the details of the new observation^17^. Similarly, vertical lines appearing in the picture (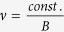, *N* = *const*.) might be associated with a basic filling factor set 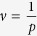.

**Figure 3:**
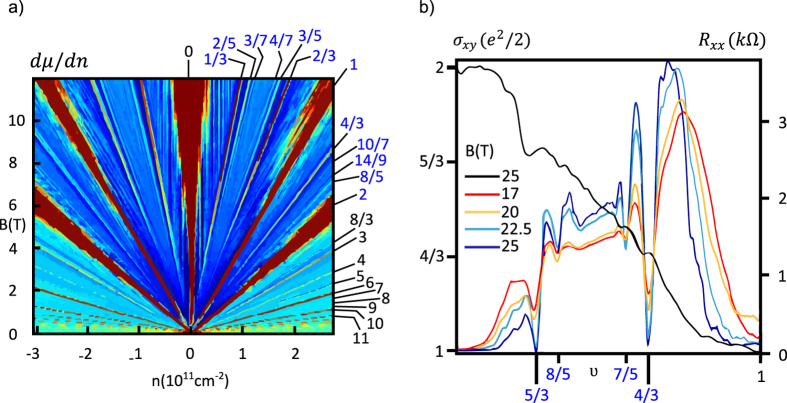
Inverse compressibility 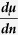 as a function of a carrier density and a magnetic field (a). 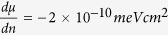
 is colored with a dark blue colour, while 
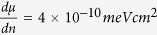
 is colored with a dark red colour. This picture is based on the measurements in ref. 12. The experimentally observed dips in the longitudinal resistance corresponding to the FQHE in the 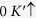 and 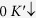 subblevels of the zeroth Landau level^24^ (**b**). The filing factors from the LLL are marked blue (there are some additional, mostly integer, filing factors from higher LLs).

**Figure 4:**
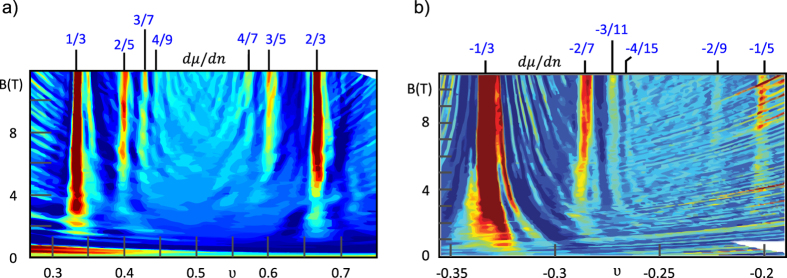
Inverse compressibility 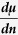 as a function of a filling factor and a magnetic field. The filling ratios from the LLL are marked blue. In the (**a**) picture 
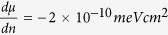
 is colored with a dark blue colour (−1 × 10^−16^ *eVm*^2^ (**b**)), while 
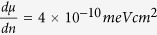
 is colored with a dark red colour (2 × 10^−16^ *eVm*^2^ (**b**)). The (**a**) diagram is based on a measurement form ref. 12, while the (**b**) diagram - from ref. 15.

The hierarchy of filling factors for the spin-valley branches 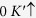 and 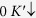 of the LLL determined by commensurability conditions stays in close agreement with the local compressibility measurements performed by Yacobi group^12^,^15^. The filling factors 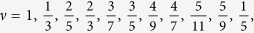 
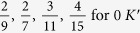
 ↑ sublevel as well as 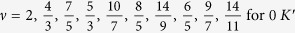 ↓ sublevel are experimentally observable and are presented in the Figs 3 and 4 (the ratios 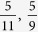 and 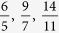 are not visible in graphs contained in this paper, but they can be easily found in Fig. 1b in ref. 15 and Fig. 4c in ref. 15, respectively).

In the next subbands – 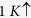, 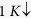, 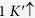 (the highest subband of the 1LL is still unreachable in experiments) – the situation slightly changes and there are three, not one, filling factors corresponding to the single-looped braid group generators (two single-loop FQHE states and one IQHE state),


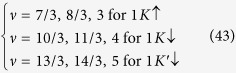


**Figure 5:**
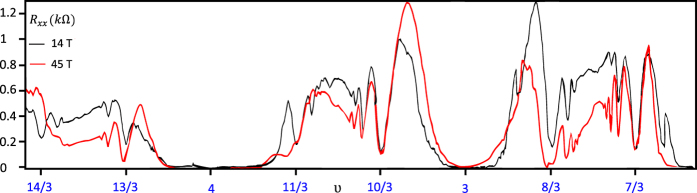
The experimentally observed dips in the longitudinal resistance corresponding to the IQHE and the sigle-loop FQHE in the 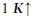, 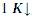, 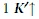 subblevels of the 1LL^24^.

All of this *ν*, presented in Fig. 5, were observed experimentally in C.R.Dean *et al.*^22^ and F.Amet *et al.*^24^ papers. Once again we emphasize that in the standard composite fermion theory integer filling factors always correspond to the IQHE^4^,^5^, while all other fractions are assigned with the FQHE. However, within the cyclotron subgroup model fractional ratios may correspond to the so-called single-loop FQHE. The collective Hall-like states, that are accomplished without the need of multi-looped trajectories, are responsible for this phenomenon. Thus, particles are not forced to traverse path consisted of many loops (to enhance a cyclotron radius and restore particle exchanges) and the system is described with a full braid group, not a subgroup. Although, these single-loop states are combined with a noninteger value of a transverse conductivity *σ*_*xy*_, its nature is expected to have more in common with the integer quantum Hall effect, rather than the ordinary FQHE. Our conclusions seem to agree with a measurment presented in Fig. 1c in ref. 24. Remarkably, the dips in a longitudinal resistance *R*_*xx*_ described with fractional filling factors 
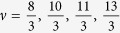
 are developing, side by side with integer *ν*, in Landau fan diagram for magnetic fields as low as 5*T*. This happens even before the famous 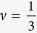 state emerges (as well as its counterpart 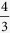 from the second spin-valley branch of the LLL). This proves that states described by multiples of 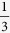 (solely in the 1LL) are almost as stable as integer quantum Hall states, as it was predicted by the topology-based model.

**Figure 6:**
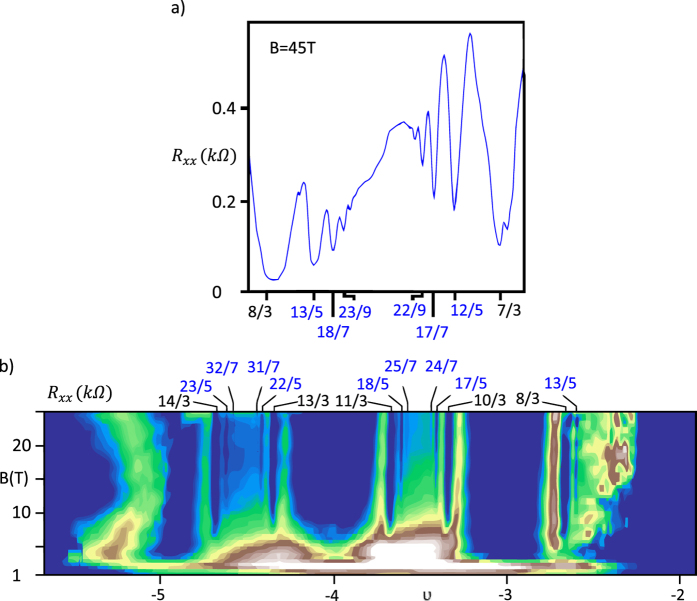
The experimentally observed values of the longitudinal resistance as a function of a filling factor (**a**) or a filling factor and a magnetoic field (**b**) in the 1LL. In the latter case 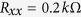 is colored with a dark blue colour, while 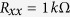 is colored with a white colour. Fractions corresponding to the FQHE in the 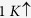, 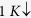 and 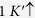 subblevels of the first Landau level are marked with blue^24^.

The FQHE filling factors from the first Landau level (determined from the main and the generalised hierarchy) are gathering close to the subband rims, which are marked with the integer filling ratios. The higher the subband index is, *ν* converge closer to the rims (this is also the feature of the FQHE fillings in typical 2D semiconductors^2^). The fractional filling factors placed in the vicinity of the IQHE ratio may disappear in its extended dip of the longitudinal resistivity. This mixing leads to the further flattening of the minimum already large in diameter. These predictions are consistent with the experimental observations, since a great amount of the theoretically derived Hall states stay beyond the resolution ability of measurement techniques. We have noticed, that only filling factors, which are separated from the nearest integer *ν* by more than 0.3 are measured. The latter is confirmed by the fact, that all filling factors – derived in the previous sections – that fulfill this condition (for example all *ν* marked with a blue colour in the Table 3),


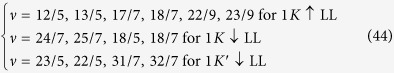


are observed in the experiments carried out in the Goldhaber-Gordon group^24^ and presented in Fig. 6.

## Discussion

The commensurability conditions are entirely based on a unique topology of two-dimensional manifolds with the magnetic field presence – and its impact on an appropriate braid group describing the multi-particle system – taken into account. In a great simplification, the commensurability conditions determine whether the cyclotron orbit fits to the interparticle distance or not. The fulfillment of fitting terms ensures that the analyzed filling factor describes the collective quantum Hall-like state. In this article we have determined the FQHE and the IQHE hierarchy for the two lowest Landau levels, which seem to stay in a perfect agreement with the data provided by experiments. It is worth to notice, that states with fractional fillings may also correspond to the single-looped fractional quantum Hall effect, when the particles do not need the multi-looped trajectories (arising from new multi-looped operators of a reduced full braid group called a cyclotron braid subgroup) for exchanges. The paper also presents a summary equations for *ν* calculation in the *n* = 0 and the *n* = 1 Landau level, but they can be easily generalized for higher LLs. We have also pointed out the basic differences between the typical 2D semiconductor and the monolayer graphene, with a special attention paid on a distinct definition of a filling factor in the latter case. It is also emphasised that, although the calculations were carried out for free electrons from the CB, the obtained hierarchy is actually a mirror reflection taken with a minus sign of the one for free holes from the VB, which is usually more pronounced in experimental results. Additionally, it is worth to remember that the specific character of the graphene energy spectrum and a nonzero Berry phase cause that the LLL is placed exactly in the Dirac point and only two out of its four sublevels are placed in the conduction band and are accessible for free electrons.

In the future work we would like to derive and present the detailed description of the appropriate braid subgroup for all filling factors, even out of the lowest Landau level. Note, that the appropriate generators (of cyclotron subgroups) were already defined for the main line 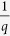 (*q* – odd) of the FQHE hierarchy^1^,^2^,^3^.

## Additional Information

**How to cite this article**: Łydżba, P. *et al.* Hierarchy of fillings for the FQHE in monolayer graphene. *Sci. Rep.* **5**, 14287; doi: 10.1038/srep14287 (2015).

## Figures and Tables

**Table 1:**
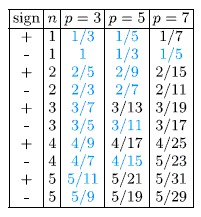
Filling factors, for the first sublevel of the LLL, for electrons manifesting the FQHE determined with the commensurability condition (multi-loop trajectories).

*The filling factors marked with a blue colour are experimentally accessible* 12,14,15,16,17,18,19,22,24. We have noticed that only fillings, which are separated from the nearest integer *ν* by more than ~0.3 − 0.2, are (at the moment) observable. Others are lost in a deep and extended minimum of a longitudinal resistivity connected with integer *ν*.

**Table 2:**
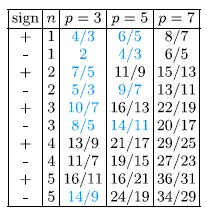
Filling factors, for the second sublevel of the LLL, for electrons experiencing the FQHE, determined with the commensurability condition (multi-loop trajectories).

*The filling factors marked with a blue colour are experimentally accessible*^12^,^15^,^17^,^22^,^24^.

**Table 3:**
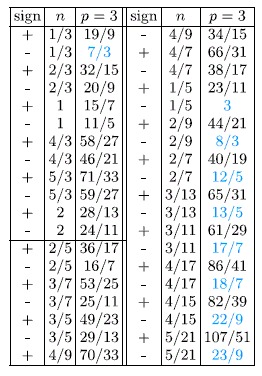
Filling factors, for the lowest sublevel of the first LL, for electrons experiencing the FQHE, determined with the commensurability condition (multi-loop trajectories).

The horizontal line separates a standard set of fractions (constructed witch *n* = *m*/3, *m*-integer) from a set of fractal filling factors (that may also be derived from higher order commensurability conditions). *Colored filling factors decribe fillings, which are separated from the nearest integer ν by more than* 0.3 *and are (at the moment) experimentally observable*^12^,^15^,^22^,^24^*. Others, are lost in a deep and extended minimum of a longitudinal resistivity connected with the integer ν.*

**Table 4:**
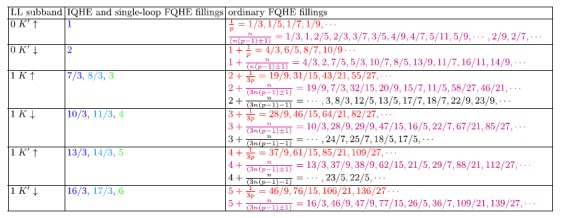
Filling factors obtained from commensurability conditions for all spin-valley branches of *n* = 0 and *n* = 1 Landau levels.

Fillings *ν* describing the IQHE and the single-loop FQHE are marked with three colours: *a dark blue colour* - the cyclotron orbit fits to the interparticle separation, *a light blue colour* - when it fits to the separation of every second particle and *a green colour* - if it fits to the separation of every third particle. Additionally, filling factors describing the ordinary FQHE are also marked with three different colours: *a red colour* - when the fraction is derived from the basic hierarchy, *a magenta colour* - when the fraction is derived from the generalized hierarchy with *n* = *m*/3 (*m*-integer) and *a black colour* - also for fractions derived from the generalized hierarchy but with *n* ≠ *m*/3 (these fillings may also be derived from higher order commensurability conditions).
